# Outcomes of polytrauma patients with diabetes mellitus

**DOI:** 10.1186/1741-7015-12-111

**Published:** 2014-07-16

**Authors:** James Tebby, Fiona Lecky, Antoinette Edwards, Tom Jenks, Omar Bouamra, Rozalia Dimitriou, Peter V Giannoudis

**Affiliations:** 1 Academic Department of Trauma & Orthopaedic Surgery, University of Leeds, Clarendon Wing, Floor A, Great George Street, Leeds General Infirmary, LS1 3EX Leeds, UK; 2 Trauma Audit and Research Network, 3rd Floor Mayo Building, Salford Royal NHS Foundation Trust, Salford M6 8HD, UK; 3 NIHR Leeds Biomedical Research Unit, Chapel Allerton Hospital, LS7 4SA Leeds, West Yorkshire, Leeds, UK; 4 LIRMM, Leeds Institute of Rheumatic and Musculoskeletal Medicine, Chapel Allerton Hospital, LS7 4SA Leeds, West Yorkshire, Leeds, UK

**Keywords:** Polytrauma, Trauma, Diabetes mellitus, TARN, Trauma Audit and Research Network

## Abstract

**Background:**

The impact of diabetes mellitus in patients with multiple system injuries remains obscure. This study was designed to increase knowledge of outcomes of polytrauma in patients who have diabetes mellitus.

**Methods:**

Data from the Trauma Audit and Research Network was used to identify patients who had suffered polytrauma during 2003 to 2011. These patients were filtered to those with known outcomes, then separated into those with diabetes, those known to have other co-morbidities but not diabetes and those known not to have any co-morbidities or diabetes. The data were analyzed to establish if patients with diabetes had differing outcomes associated with their diabetes versus the other groups.

**Results:**

In total, 222 patients had diabetes, 2,558 had no past medical co-morbidities (PMC), 2,709 had PMC but no diabetes. The diabetic group of patients was found to be older than the other groups (*P* <0.05). A higher mortality rate was found in the diabetic group compared to the non-PMC group (32.4% versus 12.9%), *P* <0.05). Rates of many complications including renal failure, myocardial infarction, acute respiratory distress syndrome, pulmonary embolism and deep vein thrombosis were all found to be higher in the diabetic group.

**Conclusions:**

Close monitoring of diabetic patients may result in improved outcomes. Tighter glycemic control and earlier intervention for complications may reduce mortality and morbidity.

## Background

Trauma itself is a pandemic which is projected to be the second most common cause of disability adjusted life years lost within the next thirteen years [1]. Trauma accounts for about 16,000 deaths a day worldwide [2]. Polytrauma represents a more severe form of trauma but currently there is no internationally standardized definition of polytrauma. Recent literature has shown improved reliability in defining polytrauma patients using an abbreviated injury score (AIS) [3] of greater than or equal to three in more than one anatomical body region compared to using the more conventional Injury Severity Scores (ISS) alone [3-5]. Death from polytrauma using AIS/ISS based data has shown an incidence in continental Europe of 25 to 50 per 100,000 per year while in Canada this number rises above 70 [2].

Much work has been done to identify how best to treat patients with polytrauma including the introduction of the universally used advanced trauma life support (ATLS) [6] system. However, no large studies have yet to look specifically at the impact of co-morbidities, and particularly diabetes, on the outcome of this group of patients. In the UK diabetes has an average prevalence of 4.45% with just fewer than three million people currently diagnosed [7]. Diabetic patients following lower extremity trauma have been shown to have an increased risk of infection and longer hospital stay [8-11]. However, the impact of diabetes in patients with multiple system injuries remains obscure. The aim of this study, therefore, is to investigate whether the presence of diabetes is associated with a higher mortality, increased length of hospital stay and/or a higher incidence of peri-operative complications (adverse outcome) following polytrauma.

## Methods

Data used were from the Trauma Audit and Research Network (TARN) database in Manchester. TARN is a non-profit organization that is funded by hospital subscriptions from England and Wales. Approximately 95% of hospitals in England and Wales are subscribed to the network and supply data about their patient populations relating to trauma. The data cover the patients’ initial presentation and subsequent inpatient stay up until their discharge. Data from each patient’s clinical record include details such as age, gender, injury scores (AIS and ISS), body area injured, Glasgow Coma Scale (GCS) score, heart rate, systolic blood pressure (SBP) on arrival, lengths of stay in hospital and complications suffered. The prospectively recorded data are then submitted to TARN and used for benchmarking/research purposes with the aim of improving acute trauma outcomes [12]. TARN already has ethical approval (PIAG section 60) for research on the anonymized data that is stored securely on the University of Manchester server. The TARN data collated over an eight year period between 2003 and 2011 were used. Patients were included if they were deemed to have suffered polytrauma. As previously discussed these patients presented with an AIS of 3 or more in two or more body regions [5]. Patients were then excluded if there was no documentation of their outcome (survival versus non-survival). Further exclusions were then applied to those whose past medical history was unclear, leaving only those who were known to have either no past medical co-morbidities or known to have co-morbidities including their diabetic status. Pre-existing co-morbidities were defined as chronic obstructive pulmonary disease (COPD), hypertension, previous myocardial infarction (MI) or renal impairment. For the purposes of data analysis patients who met the inclusion criteria were separated into three groups. Group 1 comprised patients who were known to have diabetes mellitus (DM). Where appropriate this group was further separated into those with insulin dependent diabetes mellitus (IDDM) and non-insulin dependent diabetes mellitus (NIDDM). Group 2 were patients who were known to have other past medical co-morbidities (PMC) that did not include diabetes. Group 3 patients were known to have no co-morbidities including diabetes. This process is shown in the algorithm below.

Patients were compared in the three groups in terms of their demographic and presenting physiological characteristics. Mortality in this study was defined as death within 30 days of injury from any cause within the initial hospital presentation or during any subsequent re-admission for management of the same injury. Further to all-cause mortality, the three groups were studied in more detail in order to see the incidence and mortality rates associated with a number of specific co-morbidities or complications (Table 1).

**Table 1:** Injury patterns and complications assessed for association with co-morbidity, diabetes status and outcome

**Injury patterns**	**Complications**
Head AIS 3+	Pneumonia, Wound Infection, Sepsis, UTI
Abdo AIS 3+	Renal Failure
Chest AIS 3+	MI, Arrhythmia, Arrest
Extremity AIS 3+	ARDS, DVT, PE

Abdo, abdomen; AIS, abbreviated injury score; ARDS, acute respiratory distress syndrome; DVT, deep vein thrombosis; PE, pulmonary embolism; UTI, urinary tract infection.

### Statistical analysis

The statistical analysis consisted of comparing trauma patients’ baseline characteristics, such as age, gender, ISS, and so on, among the three groups. Continuous variables, that is, age, SBP, GCS, ISS, have a skewed distribution. For our analysis we compared the medians of the three groups because of skewness (Sk). These are the values of Sk for some of the variables: GCS: Sk = -1.575 (moderate); SBP: Sk = -0.165 (slight); ISS: Sk = 0.372(slight); pulse rate: Sk = 0.042 (slight); length of stay (LOS): Sk = 1.091 (moderate); LOS ICU: Sk = 1.436 (moderate); time to operation: Sk = 3.158 (extensive); time to death: Sk = 1.019 (moderate). Due to the high number of variables with moderate to extensive skewness non-parametric techniques were used to test the null hypothesis that there is no difference in the distribution of each of the variables among the three groups. For this the Kruskal-Wallis test has been used, which is the non parametric counterpart of analysis of variance (ANOVA). For categorical variables the chi square test was used to test the hypothesis of no association between the categorical variables and the three groups. Interquartile ranges (IQR) for continuous variables are displayed as well as 95% confidence intervals for categorical variables. A *P*-value of 0.05 or below is considered statistically significant (rejecting the null hypothesis). The Statistical Package for Social Sciences (SPSS) version 16 was used to carry out the analyses.

## Results

### Group numbers

Initial results from the TARN database generated 9,629 patients between 2003 and 2011 who suffered polytrauma by our definition and had a known outcome for survival. A total of 4,140 patients were then excluded due to incomplete data regarding co-morbidities. The remaining 5,489 patients were divided into the three groups. Group 1 patients were confirmed to have diabetes mellitus, n = 222, this group was subdivided when necessary into 1a, those with non-insulin dependent diabetes mellitus (NIDDM), n = 143, and 1b, those with insulin dependent diabetes mellitus (IDDM), n = 79. Group 2 patients were those with confirmed past medical co-morbidities (PMC) but no diabetes, n = 2,709. Group 3 patients were confirmed to have no PMC and no diabetes, n = 2,558. The prevalence of diabetes in the analyzed patients was 4%.

### General observations

General analysis of the data is shown in Table 2. These results look at initial observations as well as times to theater/death/discharge. This study again shows the high percentage of males involved in trauma; this was around 70% for the diabetic and PMC groups and higher at 78% in the no PMC group.

**Table 2:** Polytrauma (AIS 3+ > =2 body regions, known outcome only) summary 2003 to 2011

**Outcome observed**	**Defined as**	**PMC**			***P*-values**
		**Diabetes**	**Known PMC no diabetes**	**Definitely no PMC**	
Gender	Female	63	858	571	<0.001
	% (95% CI)	28.4% (22.5 - 34.3)	31.7% (29.9 - 33.5)	22.3% (20.7 - 23.9)	
	Male	159	1851	1987	
	% (95% CI)	71.6% (65.7 - 77.5)	68.3% (66.5 - 70.1)	77.7% (76.1 - 78.7)	
ISS	Median ISS (IQR)	29 (25–38)	29 (22–38)	29 (22 - 38)	0.801
GCS on arrival	Median GCS (IQR)	14 (11–15)	15 (12–15)	15 (12 - 15)	0.231
SBP on arrival	Median SBP (IQR)	132 (108–154)	128 (108–146)	128 (110 - 145)	0.220
Pulse rate on arrival	Median HR (IQR)	94.5 (80–110)	93 (77–111)	95 (79 - 114)	0.058
Days in hospital	Median LOS (IQR)	17 (4–37.3)	15 (6–34)	14 (6 - 27)	0.005
Hours to death	Median time to death(IQR)	42.9 (6.8 - 130.4)	23.3 (3.7 - 125.8)	10.7 (1.9 - 95.9)	<0.001
Number of operations	Median number of operations (IQR)	2 (1–3)	2 (1–3)	2 (1–3)	0.484
Hours to operation (hours)	Median time to theater (IQR)	12.3 (3.6 - 45.8)	9.6 (3.6 - 46.9)	7.9 (3.1 - 27.5)	0.02
Critical care stay	No	83	1047	966	0.779
	% (95% CI)	37.4 (31–43.8)	38.6 (36.8 - 40.5)	37.8 (35.9 - 39.6)	
	Yes	139	1662	1592	
	% (95% CI)	62.6 (56.2 - 69)	61.4 (59.5 - 63.2)	62.2 (60.4 - 64.1)	
Days in critical care	Median LOS ICU (IQR)	3 (0–10.8)	3 (0–10)	3 (0–8)	0.147

Where appropriate values are presented in %s. AIS, Abbreviated Injury Score; CI, confidence interval; GCS, Glasgow Coma Score; HR, heart rate; IQR, interquartile range; ISS, Injury Severity Score; LOS, length of stay; PMC, past medical co-morbidities; SBP, systolic blood pressure.

There were no significant differences in the arrival observations of the patients in this review. These observations included ISS, GCS, SBP and heart rate. Injury severity expressed as an ISS was equivocal through all groups for the survivors. The ISS for non-survivors, however, was seen to be lower in both the diabetic group and the PMC group when compared to the non-PMC group (35 (Group1) versus 35 (Group 2) versus 41 (Group 3), *P* <0.05).

Diabetic patients were found to spend more time in hospital, two days longer than the PMC group and three days longer than the non-PMC group (*P* < 0.05). The LOS in ICU specifically, however, was not longer for diabetic patients.

Diabetic patients died later in their admission compared with the other groups (42.9 versus 23.3 versus 10.7, hours, *P* <0.05).

Diabetic patients were also seen to wait longer to get to theater (12.3 versus 9.6 versus 7.9, hours, *P* <0.05).

The lowest all-cause mortality was seen in patients with no PMC or DM (12.9%) and the highest seen in NIDDM patients (33.6%) and IDDM patients (30.4%), *P* = <0.05. This is shown in Table 3 along with average age of each group.

**Table 3:** Polytrauma by diabetic co morbid status (AIS 3+ ≥2 body regions, known outcome only) summary 2003 to 2011

**Sub-groups**	**Defined as**	**Diabetes**		**Known PMC no diabetes**		**Definitely no PMC**		***P*-values**
		**Outcome**		**Outcome**		**Outcome**		
		**Alive**	**Dead**	**Alive**	**Dead**	**Alive**	**Dead**	
Diabetes: Insulin dependent	No	95	48	2156	553	2229	329	N/A
	(95% CI)	66.4 (58.7 to 58.7)	33.6 (25.8 to 41.3)	79.6%	20.4%	87.1%	12.9%	
	Yes	55	24					
	(95% CI)	69.6 (59.5 to 79.8)	30.4 (20.2 to 40.5)					
Age	Median age (years)	58.1	74.3	44.5	63.6	28.6	27.7	<0.001
	IQR	42 to 70	60 to 81	29 to 61	43 to 82	20 to 43	20 to 46	

Where appropriate values are presented in %s. AIS, Abbreviated Injury Score; CI, confidence interval; IQR, interquartile range; PMC, past medical co-morbidities.

### Mechanism of injury

Tables 4 and 5 show the incidence and difference in mortality based on the mechanism of injury. Injury mechanism was investigated and separated into road traffic collision (RTC), high fall, low fall, penetrating trauma and others. Many patterns appear regarding the mortality of patients by mechanism; however, no single mechanism of injury has a statistically significant difference in mortality when comparing the three groups. Diabetic patients had a low incidence of RTC and penetrating trauma and a significantly higher incidence of fall from a height causing polytrauma.

**Table 4:** Prevalence of injury to different body regions, mechanisms of and complications from polytrauma

**Injury/Outcome**	**Present**	**PMC**			***P*-values**
		**Diabetes**	**Known PMC no diabetes**	**Definitely no PMC**	
Head AIS 3+	No	90	1174	1122	0.622
	% (95% CI)	40.5 (34.1 to 47)	43.3 (41.5 to 45.2)	43.9 (41.9 to 45.8)	
	Yes	132	1535	1436	
	% (95% CI)	59.5 (53 to 65.9)	56.7 (54.8 to 58.5)	56.1 (54.2 to 58.1)	
Abdo AIS 3+	No	197	2208	1959	<0.001
	% (95% CI)	88.7 (84.6 to 92.9)	81.5 (80 to 83)	76.6 (74.9 to 78.2)	
	Yes	25	501	599	
	% (95% CI)	11.3 (7.1 to 15.4)	18.5 (17 to 20)	23.4 (21.8 to 25.1)	
Chest AIS 3+	No	55	666	615	0.890
	% (95% CI)	24.8 (19.1 to 30.5)	24.6 (23 to 26.2)	24 (22.4 to 25.7)	
	Yes	167	2043	1943	
	% (95% CI)	75.2 (69.5 to 80.9)	75.4 (73.8 to 77)	76 (74.3 to 77.6)	
Extremity AIS 3+	No	114	1340	1248	0.719
	% (95% CI)	51.4 (44.8 to 57.9)	49.5 (47.6 to 51.3)	48.8 (46.9 to 50.7)	
	Yes	108	1369	1310	
	% (95% CI)	48.6 (42.1 to 55.2)	50.5 (48.7 to 52.4)	51.2 (49.3 to 53.1)	
Any complication	No	115	1472	1065	<0.001
	% (95% CI)	51.8 (45.2 to 58.4)	54.3 (52.5 to 56.2)	41.6 (39.7 to 43.5)	
	Yes	107	1237	1493	
	% (95% CI)	48.2 (41.6 to 54.8)	45.7 (43.8 to 47.5)	58.4 (56.5 to 60.3)	
ARDS, DVT, PE	No	210	2644	2497	0.019
	% (95% CI)	94.6 (91.6 to 97.6)	97.6 (97 to 98.2)	97.6 (97 to 98.2)	
	Yes	12	65	61	
	% (95% CI)	5.4 (2.4 to 8.4)	2.4 (1.8 to 3)	2.4 (1.8 to 3)	
Pneumonia, Wound infection, Sepsis, UTI	No	199	2483	2390	0.015
	% (95% CI)	89.6 (85.6 to 93.6)	91.7% (90.6 to 92.7)	93.4% (92.5 to 94.4)	
	Yes	23	226	168	
	% (95% CI)	10.4 (6.4 to 14.4)	8.3 (7.3 to 9.4)	6.6 (5.6 to 7.5)	
Renal failure	No	215	2671	2539	0.002
	% (95% CI)	96.8 (94.5 to 99.1)	98.6 (98.2 to 99)	99.3 (98.9 to 99.6)	
	Yes	7	38	19	
	% (95% CI)	3.2 (0.9 to 5.5)	1.4 (1 to 1.8)	0.7 (0.4 to 1.1)	
MI, arrhythmia, arrest	No	209	2590	2504	<0.001
	% (95% CI)	94.1 (91.1 to 97.2)	95.6 (94.8 to 96.4)	97.9 (97.3 to 98.4)	
	Yes	13	119	54	
	% (95% CI)	5.9 (2.8 to 8.9)	4.4 (3.6 to 5.2)	2.1 (1.6 to 2.7)	
Injury mechanism	RTC (95% CI)	117	1437	1843	<0.001
		52.7 (46.1 to 59.3)	53 (53 to 54.9)	72.0	
	High Fall (95% CI)	52	673	358	
		23.4 (17.8 to 29)	24.8 (23.2 to 26.4)	14 (12.7 to 15.3)	
	Low Fall (95% CI)	41	302	60	
		18.5 (13.4 to 23.6)	11.1 (9.9 to 12.3)	2.3 (1.7 to 2.9)	
	Other (95% CI)	10	200	188	
		4.5 (1.8 to 7.2)	7.4 (6.4 to 8.4)	7.3 (6.3 to 8.3)	
	Penetrating trauma (95% CI)	2	97	109	
		0.9 (0 to 2.1)	3.6 (2.9 to 4.3)	4.3 (3.5 to 5.1)	
Total		222	2709	2558	

Where appropriate values are presented in %s. Abdo, abdomen; AIS, Abbreviated Injury Score; ARDS, acute respiratory distress syndrome; CI, confidence interval; DVT, deep vein thrombosis; MI, myocardial infarction; PE, pulmonary embolism; RTC, road traffic collision; UTI, urinary tract infection.

**Table 5:** Mortality by body region injured, complications and mechanism of injury

**Injury/Complication**	**Present/Mechanism**	**Diabetes**		***P*-values**	**Known PMC no diabetes**		***P*-values**	**Definitely no PMC**		***P-*** **values**
		**Outcome**			**Outcome**			**Outcome**		
		**Alive**	**Dead**		**Alive**	**Dead**		**Alive**	**Dead**	
Head AIS 3+	Yes (95% CI)	74	58	<0.001	1161	374	<0.001	1163	273	<0.001
		56.1 (47.6 - 64.5)	43.9 (35.5 - 52.4)		75.6 (13.2 - 17.3)	24.4 (22.2 - 26.5)		81 (3.7 - 6.3)	19 (17 - 21)	
Abdo AIS 3+	Yes (95% CI)	19	6	0.466	373	128	0.002	523	76	0.944
		76 (59.3 - 92.7)	24 (7.3 - 40.7)		74.5 (70.6 - 78.3)	25.5 (21.7 - 29.4)		87.3 (84.6 - 90)	12.7 (10 - 15.4)	
Chest AIS 3+	Yes (95% CI)	112	55	0.911	1583	460	<0.001	1664	279	<0.001
		67.1 (59.9 - 74.2)	32.9 (25.8 - 40.1)		77.5 (75.7 - 79.3)	22.5 (20.7 - 24.3)		85.6 (84.1 - 87.2)	14.4 (12.8 - 15.9)	
Extremity AIS 3+	Yes									
(95% CI)	84	24	0.003	1114	255	0.022	1174	136	<0.001	
		77.8 (69.9 - 85.6)	22.2 (14.4 - 30.1)		81.4 (79.3 - 83.4)	18.6 (16.6 - 20.7)		89.6 (88 - 91.3)	10.4 (8.7 - 12)	
Any complication	Yes (95% CI)	69	38	0.422	956	281	0.007	1306	187	0.588
		64.5 (55.4 - 73.6)	35.5 (26.4 - 44.6)		77.3 (74.9 - 79.6)	22.7 (20.4 - 25.1)		87.5 (85.8 - 89.2)	12.5 (10.8 - 14.2)	
ARDS, DVT, PE	Yes (95% CI)	6	6	0.308	49	16	0.487	55	6	0.602
		50 (21.7 - 78.3)	50 (21.7 - 78.3)		75.4 (64.9 - 85.9)	24.6 (14.1 - 35.1)		90.2 (82.7 - 97.6)	9.8 (2.4 - 17.3)	
Pneumonia, wound infection, sepsis, UTI	Yes (95% CI)	18	5	0.357	181	45	0.913	153	15	0.145
		78.3 (61.4 - 95.1)	21.7 (4.9 - 38.6)		80.1 (74.9 - 85.3)	19.9 (14.7 - 25.1)		91.1 (86.8 - 95.4)	8.9 (4.6 - 13.2)	
Renal Failure	Yes	4	3		25	13	0.055	16	3	
					65.8%	34.2%		84.2%	15.8%	
					(50.7 - 80.9)	(19.1 - 49.3)		(60.4 - 96.6)^a^	(3.4 - 39.6)^a^	
MI, arrhythmia, arrest	Yes	4	9		41	78	<0.001	14	40	<0.001
					34.5%	65.5%		25.9%	74.1%	
					(25.9 - 43)	(57 - 74.1)		(14.2 - 37.6)	(62.4 - 85.8)	
Injury mechanism	RTC (95% CI)	85	32	0.194^a^	1173	264	0.001	1608	235	0.787
		72.6%	27.4%		81.6%	18.4%		87.2%	12.8%	
		(64.5 - 80.7)	(19.3 - 35.5)		(79.6 - 78.3)	(13.4 - 33.1)		(85.7 - 88.7)	(11.3 - 14.3)	
	High fall (95% CI)	31	21		513	160		311	47	
		59.6%	40.4%		76.2%	23.8%		86.9%	13.1%	
		(46.3 - 72.9)	(19.3 - 35.5)		(73 - 79.4)	(20.6 - 27)		(83.4 - 90.4)	(9.6 - 16.6)	
	Low fall (95% CI)	24	17		225	77		50	10	
		58.5%	41.5%		74.5%	25.5%		83.3%	16.7%	
		(43.4 - 73.6)	(26.4 - 56.6)		(69.6 - 79.4)	(20.6 - 30.4)		(73.9 - 91.9)	(7.3 - 26.1)	
	Other	8	2		159	41		162	26	
					79.5% (73.9 - 85.1)	20.5% (14.9 - 26.1)		86.2% (81.3 - 91.1)	13.8% (8.9 - 18.7)	
	Penetrating trauma	2	0		86	11		98	11	
					88.7% (82.4 - 95)	11.3% (5 - 17.6)		89.9% (84.2 - 95.6)	10.1% (4.4 - 15.8)	

Where appropriate values are presented in %s. ^**a**^*P*-value for such analyses should not be interpreted due to the small number of patients. Abdo, abdomen; AIS, Abbreviated Injury Score; ARDS, acute respiratory distress syndrome; CI, confidence interval; DVT, deep vein thrombosis; MI, myocardial infarction; PE, pulmonary embolism; RTC, road traffic collision; UTI, urinary tract infection.

### Injury distribution

For patients with a head injury of AIS ≥ 3, mortality was higher in diabetic (group 1) patients compared to group 2 and more than double that of group 3 (43.9 versus 24.4 versus 19% (group 3), *P* <0.05). The incidence of head injury was approximately 55% to 60% in all three groups.

Diabetic patients were not seen to have a difference in mortality from abdominal injuries (AIS > 3); however, the incidence was significantly lower in diabetic patients compared with the other groups (11.3 versus 18.5 versus 23.4%, respectively; *P* <0.05).

The mortality of chest injuries in diabetic patients appears to be higher than the other groups (32.9 versus 22.5 versus 14.4%, respectively); however, the comparable number of these patients in this study was insufficient to infer a global trend. The incidence of chest injuries was approximately 75% for all groups.

The mortality of patients who suffered extremity injuries of AIS ≥ 3 as part of their polytrauma was comparable, with a slightly higher mortality for the diabetic patients compared with the other groups (22.2 versus 18.6 versus 10.4%, respectively). The incidence was similar among groups at approximately 50%.

### Complications

Tables 4 and 5 also show the mortality of each group related to certain complications. The overall mortality of the diabetic group from any given complication, once developed, appears higher than the other groups (35.5 versus 22.7 versus 12.5%), respectively. However, the incidence of complications is lower in both the DM group and the known PMC group compared with the group with no PMC (48.2 versus 45.7 versus 58.4%, respectively; *P* <0.05).

There was 50% mortality for the 12 diabetic patients who developed ARDS, PE or DVT, double that of the PMC group and five times that of the no PMC group (50 versus 24.6 versus 9.8%, respectively). However, despite this trend the low number of patients seen in each group prevents us from inferring the same trends for the population parameters. A similar incidence of ARDS, DVT and PE was seen in the PMC and no PMC groups but when compared with the diabetic group, it was observed that diabetic patients are 2.25 times more likely to develop ARDS, DVT or PE (5.4 versus 2.4 versus 2.4%, respectively; *P* <0.05).

In our data sample, mortality from pneumonia, wound infection, sepsis or UTI was similar in the diabetic and PMC groups but both appeared higher than that of the no PMC group. The incidence, however, of pneumonia, wound infection, sepsis or UTI was highest in diabetic patients (10.4 versus 8.3 versus 6.6%, respectively; *P* <0.05).

Patients who developed renal failure appeared more likely to die if they were diabetic (42.9 versus 34.2 versus 15.8%, respectively). The incidence was also much higher in the diabetic group, more than double that of the PMC group and 4.5 times higher than that of the no PMC group (3.2 versus 1.4 versus 0.7%, respectively; *P* <0.05).

The mortality of patients who suffered MI, arrhythmia or arrest was found to be higher in patients in the no PMC group (69.2% versus 65.5 versus 74.1%, respectively; *P* <0.05); however, the incidence of these complications was 2.8 times higher in the diabetic patients than in the no PMC group (5.9 versus 4.4 versus 2.1%, respectively; *P* <0.05).

Table 6 shows the results of a mortality prediction model. This shows that increasing age and ISS is linked with increasing mortality. In this study, pre-existing medical conditions do not appear to cause a statistical increase in mortality. However, the presence of diabetes, even when accounting for age, ISS and GCS, does create an increase in the prediction of mortality with an odds ratio (OR) of 1.64 (*P* <0.05).

**Table 6:** Model predicting death

**Variables**	**Odds ratio**	***P*-value**	**95% CI OR**	
Age	1.06	<0.001	1.05	1.07
ISS	1.08	<0.001	1.07	1.09
GCS	0.79	<0.001	0.77	0.81
Pre-existing medical condition	1.25	0.09	0.97	1.63
Diabetes (yes)	1.64	0.026	1.06	2.53
Gender (male)	1.91	0.066	0.96	3.78
Age by gender interaction	0.99	0.107	0.98	1.00

CI, confidence interval; GCS, Glasgow Coma Score; ISS, Injury Severity Score.

## Discussion

Although many different forms of diabetes exist, the disease is generally classified as either IDDM (Type 1) or NIDDM (Type 2) [7]. These classifications have a major impact on disease progression over many years; however, for the majority of this study we have focused on diabetes as a single disease state while examining its effects over the relatively short period of treating patients who have suffered polytrauma.

The lower rate of diabetes seen in this analysis versus the UK average [7] may be explained by the higher age of diabetic patients seen in the review. Previous studies have not only shown an average age of polytrauma of around 40 but also a tendency for patients involved in polytrauma to have a younger age distribution [13-15]. It is generally taken that younger patients are also more likely to be involved in high energy accidents [13].

A mortality of more than 30% seen in diabetic patients is similar to that seen in previous studies looking at elderly patients involved in polytrauma [16] and would be consistent with the average age of diabetic non-survivors, 74.3 years. However, the age adjusted prediction model shows diabetes to be an independent predictor of increased mortality. The mortality of IDDM sufferers was also more than 30% and would seem to implicate an even higher level of relative mortality in these conversely younger patients.

Linking previous literature as well as the results of our review indicates two separate pathways leading to poor outcomes for diabetic patients. The first pathway shows a high mortality in patients who have suffered head injury AIS ≥3 in the context of polytrauma. The second pathway involves high levels of complications as well as the associated mortality that there appears to be but has not been statistically proven in this review.

It has been established that both early hyperglyaemia and insulin deficiency have been shown to increase mortality from traumatic brain injury, especially in diabetic patients [17,18]. The mortality rates in those studies, even for diabetic patients, show much lower rates of death (approximately 15% to 20%) than are observed in our results (>40%). This is likely due to the increased neurohormonal activity associated with polytrauma itself which will further increase cortisol levels and, therefore, hyperglycemia and insulin requirements [19]. This increase in blood glucose after polytrauma is not exclusive to diabetic patients suggesting that the increasing mortality seen in this group may be related to other changes associated with diabetes, such as the long term damage to the microvasculature associated with chronic diabetes [20]. In this case, the NIDDM patients would suffer more so than the IDDM patients. While there is no clear secondary mechanism for the poor outcome of diabetic patients, previous animal studies have shown that alloxan diabetes can directly change the level of vascular supply to the brain in head injuries in rat models [21]. Post mortem examinations of patients with dementia have also shown cases where untreated diabetic patients have an increased amyloid plaque load that was not seen in non-diabetic patients [22]. Identification of a reversible or treatable change in the diabetic brain may improve outcomes in this group but these changes would be part of improved secondary prevention in diabetes and not related to treating acute head injury. Certainly, these data suggest that all diabetic patients with a head injury would benefit from an insulin sliding scale with scope for recommendation that all patients who have a head injury and deranged blood glucose should have aggressive correction of hyperglycemia with insulin. Furthermore, in the acute setting, it has been shown on ICU that an increase in complications can be related to higher blood glucose levels [23-25]. This association between hyperglycemia and complications may explain why diabetic patients have a similar incidence of complications compared to the other groups despite having been involved in lower energy mechanisms. This in turn leads to the second pathway of mortality.

The second pathway leads to late deaths from increased rates of complications in diabetic patients and is separate from the mechanism of injury. The body areas affected may well dictate the types of complications expected and allow the early identification and aggressive treatment of certain injuries in diabetic patients. For example, a diabetic patient with a chest injury of AIS ≥ 3 may benefit from earlier management to prevent chest sepsis. The higher rate of complications appears in this study to be directly related to diabetes. Diabetes alone has previously been implicated as an independent risk factor for complications, especially in elderly patients in polytrauma [11]. No comment has been made previously as to which complications are more damaging or how to reduce the rates of mortality from these complications. Implementation of sliding scales to reduce hyperglycemia in ICU may reduce the rate of complications and the mortality rates seen. It is, however, likely that the majority of complications within the diabetic patient group are a result of their poor physiological status compared with other patients of similar age and injury. It has been suggested that diabetic patients have a reduced immune function compared to non-diabetic patients, especially looking at phagocytic function [26]. This may directly lead to the higher rate of infective complications, such as pneumonia and urinary sepsis, as well as the associated increased mortality. These patients may benefit from earlier intervention for the prevention of infection. For chest injuries diabetics may benefit from more intensive physiotherapy with greater pain control in order to maximize respiratory function. The earlier use of antibiotics to aid the immune system may also be beneficial. Diabetes has been shown by Kateros *et al*. to be an independent factor for developing kidney disease after orthopedic input which did not revert back to pre-morbid level [27]. This has certainly been shown to overlap into the polytrauma setting. The judicious use of nephrotoxic medicines and avoiding hypovolemia in diabetic patients may well prevent acute kidney injury and death from renal failure in this group. Diabetes is also related to increasing obesity and increased end-organ damage with these changes worsening with the duration of the patients’ diabetes [28,29]. These changes will lead to a lower physiological reserve both for survival and rehabilitation. It would appear that the most beneficial way of preventing complications and death in diabetic patients is early identification, closer monitoring and earlier intervention.

The tendency of diabetic patients to have suffered polytrauma from a low energy injury may be causing them to spend more time in emergency departments. It may only be after later assessment that these patients are identified to be suffering from polytrauma. This would provide one cause for their longer waiting times to go to theater. These delays may contribute to their increased mortality outside of the above-mentioned pathways. The increased time to death and lower rate of severe abdominal trauma or penetrating injuries (AIS ≥ 3) in the diabetic group would seem to indicate a lower rate of death by hemorrhage which would be expected to lead to a quicker death. The diabetic patients are also less likely to die quickly due to their increased tendency to have suffered polytrauma from a low energy injury, that is, low fall. Lower energy mechanisms are less likely to lead to injuries that would cause death in the acute phase. This further emphasizes that diabetic patients are more likely to develop, and die from, complications with lower energy injuries. With improved care for patients in the acute phase in hospital the death rates from lower energy injuries should be lower; the reverse, however, is seen in the diabetic group.

The aforementioned lower physiological reserve may also contribute to the diabetic patients spending longer in hospital than either of the other patient groups. This is despite spending a similar amount of time in ICU than the other groups. The increased stay on the ward is unlikely to be due solely to their increased age given that the PMC group, which also has an increased median age, did not have a similar increase in stay. The presence of diabetes would appear to be an independent factor at this stage for increasing the overall length of stay. This, in part, may be due to their increased rate of complications. However, in order to allow a step down to ward-level care these complications would need to be improving. Re-establishing safe control of blood glucose prior to discharge in diabetic patients may also be requiring extra time in hospital, although, neither of these explanations would explain the similar rates seen in LOS in ICU. This indicates the presence of a beneficial management that is occurring in the ICU environment. Certainly, the majority of studies looking at glycemic control occur within the ICU environment [23-25] and may reflect a greater monitoring of glycemic control that is not possible in the ward environment. Patients, therefore, may benefit from spending more time at higher levels of care in order to prevent longer in-patient stays.

The main strength of this study is the large numbers involved in, and the quality of the data from, the TARN database. Further strengths include the fact that the data are collected prospectively and that the data are from multiple centers within England and Wales. Multiple parameters are recorded by the centers involved and access to the database for those centers for research purposes encourages participation. The loss of 4,140 patients from the original data set of 9,629 due to incomplete documentation of pre-existing co-morbidities does create a limitation to the results and observations in the paper. Analysis of this lost group compared to those included shows that the excluded group presented with a higher ISS and had a higher mortality. They were also more likely to have suffered from a head injury. It may be that this group of patients was more likely to die earlier before their collateral history could be obtained. The percentage by year of patients lost was also examined; this showed that an average of 11% of patients had incomplete data regarding PMC. However, this number is reducing and was only 5% in 2011, therefore, having a smaller impact on the results seen.

Due to the low prevalence of diabetes, the loss of data could have led to some findings not achieving statistical significance. This is most noticeable when looking at mortality rates from complications in diabetic patients, which appear higher but have not been statistically proven. The database also relies on the centers to assess and input data correctly. This may be particularly difficult when using the AIS which already requires a subjective assessment of each patient’s injury [5]. This study is further limited by the use of a single database without incorporating data and findings of previous reviews of polytrauma patients.

In conclusion, the data analyzed have shown that diabetic patients have a mortality of more than 30% after suffering polytrauma and an increased overall mortality even when matched against age and sex. Diabetes was found to be an independent factor for increasing mortality in polytrauma, OR of 1.64, *P* <0.05. Diabetic patients are noted to have a significantly higher mortality rate from head injury in the context of polytrauma compared to the non-PMC group, OR 1.80 (1.30 to 2.51, *P* <0.05). The rate of mortality from head injury in diabetic patients in the context of polytrauma appears significantly higher than the mortality of isolated head injury in diabetic patients. Other important findings can be summarized as follows: they have a higher mortality after suffering polytrauma from a low fall versus the non-PMC group, OR = 2.49 (1.03 to 5.98, *P* <0.05); they are four times more likely to develop renal failure than patients with no-PMC, OR = 4.25 (1.77 to 10.21, *P* <0.05); they are more than 2.5 times more likely to suffer an MI, arrhythmia or arrest versus non-PMC patients, OR = 2.77 (1.49 to 5.16, *P* <0.05); they are more than two times more likely to suffer from ARDS, DVT or PE versus non-PMC patients, OR = 2.27 (1.20 to 4.27, *P* <0.05); they are nearly three times more likely to die if they suffer complications while in hospital compared to patients with no PMC, OR = 2.84 (1.90 to 4.23, *P* <0.05); and they spend on average two more ward days in hospital than other patients.

It would seem logical that the process of hyperglycemia may be responsible for some of the poor outcomes seen in diabetic patients. However, previous studies looking into glycemic control have not always focused directly on both type 1 and type 2 diabetic patients [23-25]. In order to progress from this study, a question must be asked as to how we can limit or eliminate these changes, for example, by insulin based regulation of blood sugars in all diabetic patients in ICUs and on the ward levels of complications along with the underlying chronic physiological changes in diabetic patients that make them particularly susceptible to complications from polytrauma and, as such, should be monitored closely with a low threshold for implementing early treatment.

## Conclusions

A higher rate of overall mortality and rate of complications were noted in the context of polytrauma with diabetic patients. Early identification and targeted monitoring of diabetic patients may result in improved outcomes for these patients.

## Abbreviations

AIS: Abbreviated Injury Scale; ANOVA: analysis of variance; ARDS: acute respiratory distress syndrome; ATLS: advanced trauma life support; BP: blood pressure; COPD: chronic obstructive pulmonary disease; DM: diabetes mellitus; DVT: deep vein thrombosis; GCS: Glasgow Coma Scale; ICU: intensive care unit; IDDM: insulin dependent diabetes mellitus; IQR: interquartile range; ISS: Injury Severity Score; LOS: length of stay; MI: myocardial infarction; NIDDM: non-insulin dependent diabetes mellitus; OR: odds ratio; PE: pulmonary embolism; PMC: past medical co-morbidities; RTC: road traffic collision; Sk: skewness; TARN: Trauma Audit and Research Network; UTI: urinary tract infection.

## Competing interests

The authors declare that they have no competing interests.

## Authors’ contributions

PG and FL conceived of the study, and participated in its design and coordination and helped to draft the manuscript. JT was responsible for the manuscript. AE, TJ and OB provided statistical analysis from the database. RD helped draft the manuscript. All authors read and approved the final manuscript.

## Pre-publication history

The pre-publication history for this paper can be accessed here:

http://www.biomedcentral.com/1741-7015/12/111/prepub
